# The balance of intrapersonal, interpersonal, and extra-personal values and its relationship to life satisfaction and resilience in Japan and the United States

**DOI:** 10.3389/fpsyg.2025.1606618

**Published:** 2025-07-30

**Authors:** Aiko Murata, Miki Yokoyama, Wataru Akahori, Junji Watanabe

**Affiliations:** ^1^Communication Science Laboratories, NTT, Inc., Atsugi, Japan; ^2^Social Informatics Laboratories, NTT, Inc., Yokosuka, Japan

**Keywords:** well-being, balance of values, life satisfaction, resilience, cross-cultural survey

## Abstract

Previous studies have shown that the values essential to well-being vary across individuals and cultures. Most cross-cultural well-being studies have focused on cultural differences related to the importance of specific values such as achievement and universalism. Recently, however, some researchers focusing on East Asian cultures have noted the importance of wholeness, integration, and balance in well-being, where individual, social, and environmental factors are harmoniously integrated. The current study focuses on the balance individuals strike among each of the following values: (1) intrapersonal values, which they fulfill personally; (2) interpersonal values, which they fulfill in their relationships with others; and (3) extra-personal values, which they fulfill in their relationships with society as a whole and the greater world. Specifically, two aspects of the balance of values: (A) Value Diversity: the extent of diversity in value selection by identifying how many of the three predefined value categories were chosen, (B) Value Proportion: the relative emphasis placed on each value category by identifying which of the three value categories was selected more frequently. Using a large-scale online survey, we explored the relationship between the balance of values and life satisfaction (satisfaction with one’s current life) and resilience (the tendency to recover from negative events) among participants in Japan (*n* = 5,219) and the United States (*n* = 4,818), spanning various age and socioeconomic groups. The results indicated that, regardless of country, individuals with greater Value Diversity exhibited higher resilience. Those who placed greater emphasis on extra-personal values also tended to be more resilient. In Japan, but not in the United States, a stronger emphasis on extra-personal values was associated with higher life satisfaction. These findings suggest that while the relationship between Value Proportion and life satisfaction differs across cultures—being observed in Japan but not in the United States—the balance of multiple values, rather than reliance on a single value type, appears to contribute to the resilience that supports future well-being in both countries, and thus not only in East Asian cultures.

## 1 Introduction

Well-being is considered one of the key goals in the Sustainable Development Goals (SDGs) adopted by the United Nations in 2015, and surveys on well-being have accordingly been conducted by groups in many countries, including the Organization for Economic Co-operation and Development (2020) and World Happiness Report (e.g., Helliwell et al., 2025). The concept of well-being is widely accepted by the general public, and attention to research on well-being has increased over the years. The factors that contribute to well-being vary from person to person. For some, it is important to be able to achieve goals, whereas for others, it is important to spend time with their families and loved ones, or to feel connected to the natural world. Providing well-being support that aligns with individual needs requires an understanding of what is important to each person’s well-being—in essence, their core values.

Previous studies have examined the relationship between subjective well-being and what an individual values. For example, Sagiv and Schwartz (2000) conducted a survey of people in Germany and Israel and reported that among Schwartz’s ten basic values—Power (having economic and social influence), Achievement (achieving success and being recognized for it), Hedonism (experiencing pleasure and enjoyment), Self-direction (being creative and acting independently), Universalism (caring for the environment and nature), Stimulation (leading an adventurous and exciting life), Benevolence (caring for the welfare of others and society), Tradition (adhering to cultural customs and traditions), Conformity (behaving in a polite and socially appropriate manner), and Security (prioritizing safety and avoiding risks; see Schwartz, 1992)—Achievement, Self-direction, and Stimulation were positively correlated with short-term affective well-being (positive/negative affect experienced in the past few weeks), whereas Tradition, Conformity, and Security were negatively correlated with short-term affective well-being. No significant correlations were found with long-term cognitive well-being, such as life satisfaction. Joshanloo and Ghaedi (2009) also examined the relationship between Schwartz’s ten basic values and psychological and social well-being (Ryff and Keyes, 1995; Keyes, 1998), in addition to affective well-being and life satisfaction. Their findings showed that Achievement was positively correlated with psychological well-being, life satisfaction, and affective well-being, and Tradition was negatively correlated with psychological and affective well-being. Power and Self-direction were negatively correlated with social well-being, and Benevolence and Conformity were positively correlated with social well-being.

Several studies have reported that the values important to well-being vary across cultures and social situations (e.g., Oishi et al., 2009; Sortheix and Lönnqvist, 2014; Uchida and Ogihara, 2012). For example, Sortheix and Lönnqvist (2014) reported that the relationship between Achievement and life satisfaction is positively correlated in countries with low socioeconomic development but negatively correlated in countries with high socioeconomic development. They also found that the relationship between Universalism and life satisfaction was positively correlated in countries with high socioeconomic development but negatively correlated in countries with low socioeconomic development. Uchida and Ogihara (2012) pointed out that in European-American cultural contexts, individuals tend to pursue happiness as autonomous agents, with personal goal attainment and high self-esteem or self-efficacy being the major factors underlying happiness. In contrast, in East Asian cultural contexts, individuals tend to view themselves as interdependent with others and society, with harmony in social relationships and emotional support from others being the major factors in determining happiness.

While many studies have focused on the importance of specific values for people’s well-being, some researchers have recently noted that a sense of wholeness, integration, and balance or harmony is also important to well-being (Lomas et al., 2022a; Lomas et al., 2022b). For example, research has shown that individuals with a higher sense of life balance—measured by the question, “In general, do you feel the various aspects of your life are in balance, or not?”—tend to have higher life evaluation scores (i.e., how positively they assess their own lives). While this relationship is observed in “WEIRD” (Western, Educated, Industrialized, Rich, and Democratic, see Henrich et al., 2010) countries as well, it is particularly strong in East Asian countries (Lomas et al., 2022b).

These researchers focus on a subjective “sense of balance,” while other researchers highlight the balance between personal concerns, those of close others, and broader societal and global matters. In particular, Swaminathan et al. (2021), referring to traditional Indian philosophy, noted that in East Asian cultures, a good balance has long been considered important for well-being, where individual and social characteristics are realized together. The balance between the individual and the ecosystem as a whole—that is, the balance between matters concerning the individual, those concerning others, and those concerning society and the world—has been emphasized. The World Health Organization (WHO) also stated that “Mental health is a state of mental well-being that enables people to cope with the stresses of life, realize their abilities, learn well and work well, and contribute to their community. It is an integral component of health and well-being that underpins our individual and collective abilities to make decisions, build relationships and shape the world we live in” (World Health Organization, 2022). The WHO explicitly pointed out that not only the values exhibited by one person but also interpersonal/extra-personal values, such as the relationships with others and society, are pivotal for well-being.

In the current study, using the above arguments as a framework, we focus not on the subjective “sense of balance” but rather on “the balance of values” between individual values and values related to connections with others, society, and the world, and examine their relationship to well-being. Specifically, we investigate two aspects of the balance of values: (A) Value Diversity, the extent to which individuals prioritize a diverse range of values across the three categories classified from the perspective of social relatedness; and (B) Value Proportion, the relative emphasis individuals place on each category of values. We then examine how these aspects relate to subjective well-being. Given that the relationship between specific values and subjective well-being can vary across cultures (e.g., Oishi et al., 2009; Uchida and Ogihara, 2012; Sortheix and Lönnqvist, 2014), we also assess whether this relationship differs between cultures.

In a study on how people can use technology to support well-being enhancement, Calvo and Peters (2014) categorized the factors essential to a person’s well-being into three values based on the scope of social relationships. First, intrapersonal values, such as a sense of immersion and achievement, can be felt alone. Positive emotion is a typical intrapersonal value and includes momentary emotions such as joy and pleasure. Continued mental activity, such as immersion and mindfulness, are also intrapersonal values, as are values related to self-recognition, such as self-esteem, autonomy, achievement, and competence. Second, interpersonal values, such as good relationships, empathy, and trust, can be experienced in relation to others. It has been reported that gratitude (Emmons and McCullough, 2003) and empathy (Morelli et al., 2015) influence well-being. Third, extra-personal values, such as social responsibility, humility and spirituality, can be realized in a world beyond specific relationships with others (see Calvo and Peters, 2014). The mindset of accepting the world beyond the self, and harmony in social relationships and the ecosystem as a whole (Swaminathan et al., 2021), are considered to be among these values.

In light of these arguments, we focus on intrapersonal values, interpersonal values, and extra-personal values and investigate the balance between them. The points made by Swaminathan et al. (2021) raise several questions, as follows. Is the balance of values that has historically been emphasized in East Asian culture particularly important in the East Asian sphere? Or, is this balance also relevant in European-American culture, given that the WHO also points to the importance of relationships with society and the world (World Health Organization, 2022)? To examine which of the three types of values are related to well-being, and whether a balance of these values is important for well-being across cultures, we conducted a cross-cultural survey in Japan, as a representative country of the East Asian cultural sphere, and the United States (US), as a representative country of the European-American cultural sphere.

We prepared a list of diverse values in three categories—intrapersonal, interpersonal, and extra-personal—(Supplementary Table 1) on the basis of the responses of a previous survey that asked respondents to write freely about the values that affect their own well-being (Watanabe and Chen, 2021; JST RISTEX HITE, 2019). An explanation of the process of preparing a list of diverse values is presented in the Methods section. We conducted a large-scale cross-cultural online survey using the list to test the hypothesis that a balance of values is associated with well-being. Before evaluating the balance of values for each person, we checked which items (specific values) were most likely to be selected in each value category in the US and Japan. Then, to evaluate the balance of values, we examined the following indicators:

(A) Value Diversity: This indicator captures the extent of diversity in value selection by identifying how many of the three predefined value categories were chosen—specifically, whether the respondent selected values from one, two, or all three categories.
(B) Value Proportion: This indicator captures the relative emphasis placed on each value category by identifying which of the three value categories was selected more frequently—specifically, the proportion of intrapersonal, interpersonal, and extra-personal values selected.

We then explored how the two aspects of the balance of values—Value Diversity and Value Proportion—were related to subjective well-being.

We utilized indices that assess long-term subjective well-being rather than short-term affective well-being so as to focus on the relationship between the balance of values and relatively sustained well-being. Life satisfaction is one of the best-known indicators used to assess long-term subjective well-being (Diener et al., 1985), which measures how satisfied a person is with their life. It should be noted that, to maintain a state of well-being, it is important not only to be “satisfied with the situation one is in” but also to be able to “bounce back from difficulties” (i.e., resilience). A typical definition of resilience is “the process, ability, and outcome of successfully adapting to difficult and threatening situations” (Masten et al., 1990). Many studies have suggested that resilience is essential for well-being (see Harms et al., 2018, for a review). Thus, we examined how the balance of values relates to life satisfaction and resilience in the US and Japan.

## 2 Materials and methods

### 2.1 Participants

The online survey was conducted from March 12-21, 2021 with participants whose country of birth and place of residence was either the US or Japan. The demographic characteristics of participants are listed in Supplementary Table 2. In the US, demographic information and subjective well-being was obtained in addition to responses on values during this survey. In Japan, the survey on values was conducted on monitors stored in the Human Information Database® (NTT Data Institute of Management Consulting, Inc.), which already contained records for subjective well-being scores collected in August 2020.

The study protocol was approved by the NTT Communication Science Laboratories Research Ethics Committee (R02-016) and was performed in accordance with the ethical standards set down in the 2013 Declaration of Helsinki. Informed consent was obtained from all participants using a web form before starting the questionnaire. Participants were presented with an explanatory document outlining the purpose and procedures of the survey, as well as the publication of survey data. It was also stated that the collected data would be managed using anonymized IDs, ensuring that participants’ personal information would not be used or disclosed. Only individuals who consented by clicking the “Agree” button participated in the survey. Upon completing all responses, participants received compensation (i.e., points) in accordance with the survey company’s terms and conditions.

### 2.2 A list of diverse values based on previous research

In the present study, to reduce the psychological burden on participants, instead of using a free-text format, we provided participants with a list of diverse values prepared with reference to the responses in the previous survey (Watanabe and Chen, 2021; JST RISTEX HITE, 2019) and asked them to select the values they felt were important to them. The previous survey was conducted by asking students from multiple Japanese universities to describe in free-form writing the three values that they thought contributed most to their well-being. On the basis of the free-text responses, two of the authors who are trained psychologists discussed the list of values to be used in the present study and eliminated the overlap between items by combining similar items into one. The categorization of values in this study was based on the perspective of social relatedness. While this approach is similar to the axes of individual achievement orientation and relationship orientation outlined by Uchida and Ogihara (2012), as well as the distinction between self-enhancement and self-transcendence in Schwartz’s (1992) framework, the present study drew on Calvo and Peters (2014) to classify values into three categories: (1) intrapersonal values, which are fulfilled personally; (2) interpersonal values, which are fulfilled in relationships with others; and (3) extra-personal values, which are fulfilled in relationships with society as a whole and the broader world. Time and financial resources, eating, and sleeping were not included in the list of items because they were not considered appropriate for examining individual differences in the balance of values, as these are important issues for almost everyone if they are severely lacking. We then added “Leadership” (Kuoppala et al., 2008), “Awareness of human kindness” (referred to as “Elevation” in Haidt, 2000), and “Prayer” (Whittington and Scher, 2010), all of which have been reported to be related to well-being in previous studies. Finally, we prepared 16 to 22 items each for intrapersonal, interpersonal, and extra-personal values (Supplementary Table 1).

### 2.3 Two-stage selection method

In large-scale surveys such as the World Value Survey, it is generally preferable to ask a small number of questions, such as the 10-item scale of Schwartz’s ten basic values (Schwartz, 1992), to avoid increasing the burden on participants. However, the quantitative method for measuring such a small number of items on a Likert scale has limitations in terms of capturing the diversity of each individual’s values. Researchers have highlighted the importance of combining both quantitative and qualitative methods to study variation in well-being among individuals (Delle Fave et al., 2011). Studies have utilized an open-ended response method to identify values that contribute to subjective well-being, which were then divided into categories and co-occurring terms to examine their relationship to demographics and culture (e.g., Lu and Gilmour, 2006; Kobayashi and Hommerich, 2018). Therefore, to capture the diverse values of each individual in a more specific and high-resolution manner while reducing the burden on respondents, we implemented a method in which respondents select from multiple items indicating specific values created on the basis of free-text responses obtained in a previous qualitative survey (Watanabe and Chen, 2021; JST RISTEX HITE, 2019).

**Figure 1:**
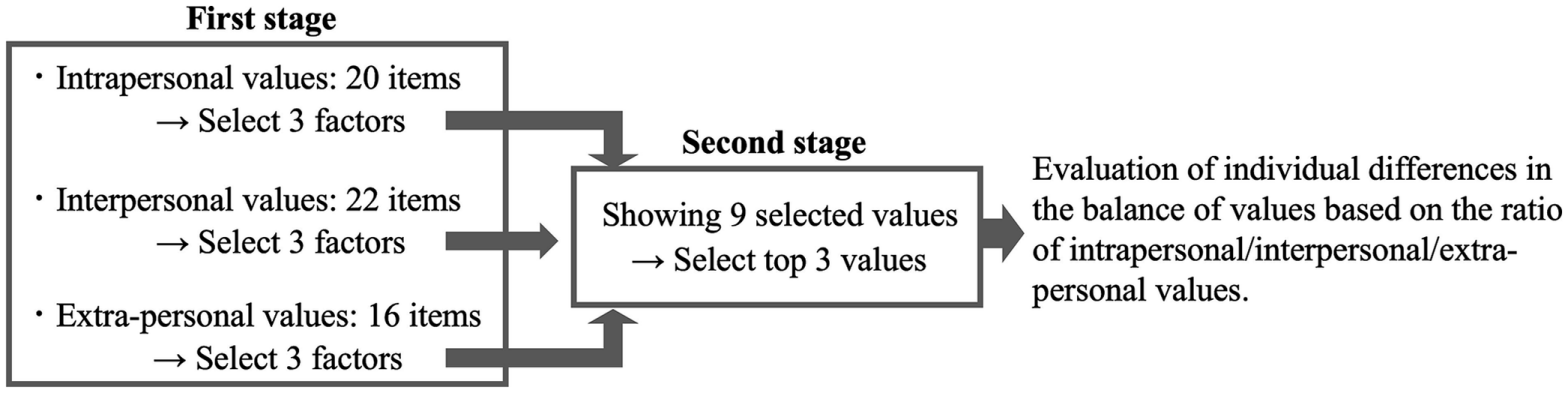
Two-stage selection method utilized in this study. In the first selection stage, participants were presented with the following statement and asked to select the values they considered to be important in their lives: “According to you, what is important in life? Please select three items from each of categories A to C that you consider to be important (total of nine).” Intrapersonal values were presented in Category A, interpersonal values in Category B, and extra-personal values in Category C (see Table 1 and Supplementary Table 1 for the list of specific descriptions of all values). In the second selection stage, for the nine values selected in the first selection stage, participants were presented with the statement, “Among the nine items that you selected, please select three that you consider to be particularly important”.

As described above, we prepared 20, 22, and 16 items in each of the three categories, for a total of 58 items. However, to address the concern that showing all items at the same time would increase the psychological burden because of an excessive number of options (see Iyengar, 2010), we designed a two-stage selection method: (1) select within the three categories and then (2) re-select the most important items among those selected. In the first selection stage, respondents were asked to select three values from each of the three categories. In this stage, the order in which the value items were presented within each category was randomized for each participant. In the second selection stage, participants were asked to select three values that they felt were particularly important to them from the total of nine values selected in the first selection stage (see Figure 1). This approach allowed for correcting the bias in selection probabilities between categories caused by the number of items in each category being different (i.e., 20, 22, and 16 items).

The two-stage selection process allowed participants to further evaluate the relative importance of the values that they selected as important in each category, enabling them to carefully weigh and select the items that best represent their own beliefs. From the perspective of the researcher, analyzing the final three values selected provides insight into the balance of the proportions of intrapersonal, interpersonal, and extra-personal values for each participant.

### 2.4 Questionnaire of subjective well-being

Since this study specifically focuses on sustainable long-term well-being rather than temporary affective well-being, we used life satisfaction (the degree of satisfaction with one’s current life) and resilience (the tendency to recover from a negative event) as indices for evaluating subjective well-being.

#### 2.4.1 Life satisfaction

We used the Satisfaction with Life Scale (Diener et al., 1985), which consists of the following five items: “1. In most ways my life is close to my ideal,” “2. The conditions of my life are excellent,” “3. I am satisfied with my life,” “4. So far I have gotten the important things I want in life,” and “5. If I could live my life over, I would change almost nothing.”

Although the scale reliability for the Japanese survey cannot be calculated because we used scores stored in the database, the scale reliability for the US survey was sufficiently high (Cronbach’s alpha = 0.90). The questions were answered from 1 (strongly disagree) to 7 (strongly agree) and the total score was calculated to create a single score (i.e., scores range from 5 to 35).

#### 2.4.2 Resilience

The Psychological Resilience Scale (Oshio et al., 2002; Oshio et al., 2003) was used for assessing resilience. This scale consists of 21 items on the following three dimensions: “Novelty Seeking” (e.g., I like new or intriguing things), “Emotional Regulation” (e.g., I can stay calm in tough circumstances), and “Positive Future Orientation” (e.g., I feel positive about my future). This scale, available in both Japanese and English, measures individual differences in psychological resilience. While it was originally developed primarily for adolescents, it assesses general resilience in the face of difficulties regardless of age, and has also been used in studies involving adults (e.g., Gito et al., 2013; Tobe et al., 2019).

Although the scale reliability for the Japanese survey cannot be calculated because we used scores stored in the database, the scale reliability for the US survey was sufficiently high (Cronbach’s alpha = 0.86 for all items). The participants responded using a five-point scale ranging from 1 (definitely no) to 5 (definitely yes). Higher scores indicated higher resilience. The mean score of 21 items was calculated for the analysis.

### 2.5 Statistical analyses

For the data in the first selection stage, in order to clarify which values are most likely to be selected in the intrapersonal, interpersonal, and extra-personal categories in the US and Japan, the frequency of selection of each value in each category was calculated and compared using the chi-squared test. For the data from the second selection stage, we calculated two aspects of the balance of values: (A) Value Diversity, defined as the number of value categories selected (one, two, or all three), and (B) Value Proportion, defined as the selection rate of each of the three value categories. Differences between countries were examined using Welch’s two-sample t-test for Value Diversity and a chi-square test for Value Proportion. Finally, we conducted linear regression analyses to control for the effects of demographic characteristics on life satisfaction and resilience scores, with the aim of examining whether life satisfaction and resilience differ according to the balance of values—Value Diversity and Value Proportion—and whether this relationship differs between the US and Japan. To avoid multicollinearity concerns due to the structural relationship between Value Diversity and Value Proportion, we constructed separate regression models for each indicator. In this study, because of the large sample size, we used the Bayesian Information Criterion (BIC: Schwarz, 1978) as the criterion for selecting the optimal model. However, we also included the best-fit model based on the Akaike Information Criterion (AIC: Akaike, 1974), which is often used even with relatively small sample sizes, in the Supplementary results for reference (Supplementary Tables 5, 6).

## 3 Results and discussion

### 3.1 Frequency of selection of each value in the first selection stage in the US and Japan

First, for the first selection stage of responses, we calculated the frequency of selection of each value in each category to determine which values were most likely to be selected in the intrapersonal, interpersonal, and extra-personal categories in the US and Japan (Table 1). We performed a chi-square test to examine the effect of country on the pattern of value selection rates for each category. For the intrapersonal category, there are differences in the value selection rates between the US and Japan (*χ*^2^_19_ = 1309.5, *p* < 0.001, Cramer’s V = 0.209). Specifically, while “immersion in interest” was frequently selected in both the US and Japan, “tenacity,” “hope,” and “keeping your own pace” were more likely to be selected in the US, and “self-determination,” “mindfulness,” and “curiosity” were more likely to be selected in Japan. For the interpersonal category, there are also differences in the value selection rates between the US and Japan (*χ*^2^_21_ = 2380.3, *p* < 0.001, Cramer’s V = 0.281). “Close relationships” was frequently selected in both the US and Japan, while “to love others” and “to be loved” were more likely to be selected in the US, and “trust,” “maintaining boundaries,” and “mutual gratitude” were more likely to be selected in Japan. For the extra-personal category, there are also differences in the value selection rates between the US and Japan (*χ*^2^_15_ = 2226.4, *p* < 0.001, Cramer’s V = 0.272). Specifically, while “diversity” and “gratitude for life” were commonly selected in both Japan and the US, “prayer” and “contributing to society” were more likely to be selected in the US, and “peace” and “following social norms” were more likely to be selected in Japan.

**Table 1:** Selection ratios for each category of values in the US and Japan.

Intrapersonal values	US	Japan	Interpersonal values	US	Japan	Extra-personal values	US	Japan
Immersion in interests	8.10%	9.60%	Close relationships	9.90%	10.80%	Trustworthy society	5.40%	6.90%
Goal-oriented	5.40%	5.60%	Community relations	3.40%	3.80%	Peace	7.10%	11.50%
Hope	6.70%	5.00%	Shared experiences with family/friends	7.50%	7.20%	Diversity	11.00%	12.30%
Tenacity	8.60%	6.40%	Shared experiences with the community	3.60%	1.70%	Contributing to society	7.80%	4.30%
Keeping your own pace	6.50%	4.80%	Building new relationships	3.00%	2.30%	Role in society	5.20%	5.50%
Mindfulness	5.80%	8.40%	Cooperation	2.90%	2.90%	Following social norms	3.70%	8.70%
Self-awareness	2.90%	5.00%	Achieving victory	2.00%	1.20%	Acknowledging the unknown	4.00%	6.40%
Self-acceptance	4.90%	5.90%	Harmony	2.20%	4.30%	Being a part of nature	7.50%	5.40%
Self-determination	4.20%	9.20%	Acceptance	3.70%	5.60%	Gratitude for life	9.80%	9.70%
Sense of competence	6.00%	2.50%	Self-esteem	3.90%	2.60%	Awareness of mortality	2.70%	5.30%
Sense of accomplishment	6.30%	5.20%	Altruism	6.00%	3.70%	Awareness of human kindness	6.70%	6.00%
Personal growth	4.40%	4.20%	Trust	6.20%	10.60%	Balancing conflicting views	5.20%	5.30%
Challenging oneself	6.20%	3.50%	To love others	9.20%	4.30%	Prayer	9.00%	1.40%
Fulfilling one’s potential	3.70%	3.90%	To be loved	9.00%	6.20%	Observance of religious precepts	4.70%	1.10%
Vitality	5.20%	4.30%	Mutual gratitude	3.40%	7.80%	Equitable society	5.10%	7.00%
Uncompromisingness	1.60%	3.30%	Having good role models	2.70%	1.50%	Ancestral bonds	5.10%	3.00%
Curiosity	4.80%	6.90%	To be respected	6.00%	1.90%	Total	100%	100%
Error avoidance	2.00%	3.10%	Mutual empathy	3.10%	5.10%			
To be the best	3.00%	1.10%	To have fun with everyone	3.90%	2.30%			
Uniqueness	3.70%	2.10%	Maintaining boundaries	1.70%	8.00%			
Total	100%	100%	Shared values	4.30%	4.90%			
			Leadership	2.60%	1.40%			
			Total	100%	100%			

Gray shading indicates the top four values with the highest selection rates in each country.

Overall, the difference in the selection ratio between the US and Japan was less than 3% for approximately 80% of the value items, which suggests some commonalities in the values considered important across cultures, as also noted in previous research (Schwartz, 1992). Furthermore, there were no items with extremely high or low selection rates in both the US and Japan, and even items with low selection rates were selected by more than 50 people. While this observation is descriptive in nature and does not replace formal statistical testing, it supports the usability of the list we utilized in the present study to capture, to some extent, the diverse values that varied among individuals in both countries.

Notable differences were found between the selection rates of the two countries, especially for “maintaining boundaries,” which had a high selection rate in Japan, and “prayer,” which had a high selection rate in the US. Regarding “maintaining boundaries,” a previous study did not directly compare Japanese and US preferences for social distance but showed that the preferred interpersonal distance was closer in the US compared to that in South Korea and China (Sorokowska et al., 2017), suggesting that the importance of social distance from others may be greater in East Asian cultural areas. Regarding “prayer,” this may reflect differences in religion: prayer is common in the US, where Christianity is the main religion, but may be less common in Japan, where Shintoism and Buddhism are the main religions. Also, given that Japanese people tend to have low religious affiliation—with only 16% self-identifying as religious (WIN-Gallup International, 2012)—it is likely that prayer was less frequently selected as an important personal value.

### 3.2 The balance of the selected categories in the second selection stage in the US and Japan

Next, we calculated Value Diversity by determining how many of the three predefined value categories each respondent selected—that is, whether their selected values spanned one, two, or all three categories. Value Diversity thus ranged from 1 to 3. A Welch’s two-sample t-test revealed significantly greater Value Diversity in the US (*M* = 2.70, SD = 0.56) than in Japan (*M* = 2.62, SD = 0.57), t_10004_ = 7.52, *p* < 0.001, Cohen’s d = 0.15, 95%CI [0.11, 0.22]. These findings suggest that, compared to Japan, individuals in the US are more likely to endorse multiple values across the intrapersonal, interpersonal, and extra-personal value domains.

For Value Proportion—which reflects the relative emphasis placed on each value category by identifying which of the three categories was selected more frequently—we calculated the proportions of intrapersonal, interpersonal, and extra-personal responses during the second selection stage (Table 2). Although the extra-personal category had a slightly lower selection rate, the proportions across all three categories ranged from approximately 0.30 to 0.35 and did not differ substantially. The results of the chi-square test revealed that the selection ratio for each category was slightly different between the US and Japan (*χ*^2^_2_ = 11.258, *p* = 0.004, Cramer’s V = 0.019). The selection ratio of intrapersonal was higher in the US compared to that in Japan, and the selection ratio of interpersonal and extra-personal was higher in Japan compared to that in the US. Although the difference in the proportions of intrapersonal, interpersonal, and extra-personal values between the US and Japan is not substantial, this finding that intrapersonal values are more likely to be emphasized in the US while interpersonal and extra-personal values are more likely to be emphasized in Japan is consistent with research in cultural psychology (e.g., Uchida and Ogihara, 2012). This research shows that in European and American cultures, which view individuals as autonomous agents, values that can be achieved individually are associated with happiness, whereas in East Asian cultures, which view individuals as interdependent with others and society, values that can be achieved in social relationships are associated with happiness.

**Table 2:** Proportion of each value category selected.

Category	US	Japan
Intrapersonal values	0.364	0.346
Interpersonal values	0.338	0.347
Extra-personal values	0.298	0.308
Total	1.000	1.000

### 3.3 Relationship between demographic characteristics, life satisfaction, and resilience

We conducted linear regression analyses to examine the effects of demographic characteristics on life satisfaction and resilience scores, prior to the main analyses. Age, gender, range of family income, marital status, and country were entered into the linear regression model as fixed effects. Similar to the findings of a previous study (Oishi et al., 2009), the results revealed that life satisfaction scores were lower in Japan than in the US. In addition, life satisfaction was higher for those who were married compared to those who were not (including those who were separated, divorced, or widowed), and there were no differences in life satisfaction by gender. Regarding age groups, life satisfaction for individuals in their 20s and 60s was similar, while for those in their 30s to 50s, it was lower, and life satisfaction was higher for individuals with higher family income (see Supplementary Figure 1). Resilience scores were also lower in Japan than in the US. Men showed slightly higher resilience than women, and the resilience score increased according to the level of family income and age. The resilience score was also slightly higher for participants who were married compared to those who were not (Supplementary Figure 2).

### 3.4 Relationship between value diversity and life satisfaction

Next, we conducted a linear regression analysis to determine whether life satisfaction varied by Value Diversity, and whether the relationship differed between the US and Japan. Factors relevant to our hypotheses were the effect of Value Diversity and the interaction effect between Value Diversity and country. To control for demographic factors, we included them as covariates in the model. Age, gender, range of family income, marital status, country, Value Diversity, and the interaction effects between Value Diversity and country were entered into the linear regression model as fixed effects. Given the large sample size of the present study, models with all combinations of fixed effects were fitted, and their goodness of fit was compared using BIC.

The best-fit model contained only the main effects of demographic factors, age, family income, marital status, and country, and did not include the effects of Value Diversity or the interaction between Value Diversity and country. This suggests that Value Diversity is not related to life satisfaction in either the US or Japan.

### 3.5 Relationship between value diversity and resilience

Next, we conducted a linear regression analysis to determine whether resilience scores varied by Value Diversity, and whether the relationship differed between the US and Japan. The fixed effects included in the model were identical to those in the model for life satisfaction, namely, the demographic characteristics, Value Diversity, and the interaction effects between Value Diversity and country. The models for all possible combinations of fixed effects were fitted and compared in terms of the degree of fit according to the BIC.

**Figure 2:**
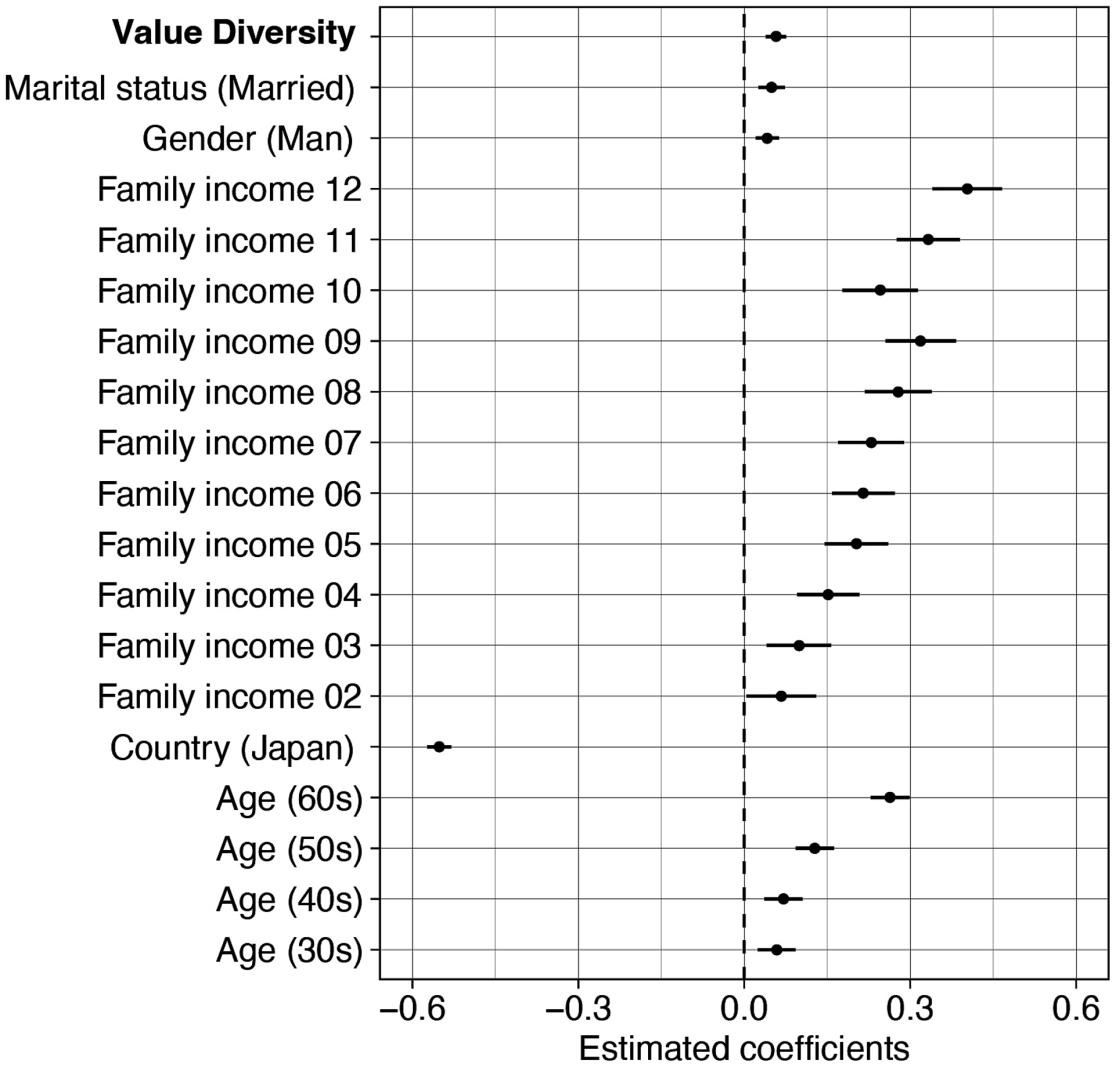
Parameter estimates of the best-fit model for resilience with the effect of Value Diversity based on BIC and AIC. Linear regression models comparing the effects of Value Diversity, with demographic variables included as covariates. Error bars represent 95% confidence intervals of estimates.

The best-fit model contained the main effects of age, gender, family income, marital status, country, and Value Diversity, but no Value Diversity × country interaction. Parameter estimates of the best-fit model are shown in Figure 2 and Supplementary Table 3. In the best-fit model, the effect for the Value Diversity (*B* = 0.057, 95%CI [0.038, 0.075], t_10017_ = 6.045, *p* < 0.001) was greater than 0. This indicates that, regardless of country, individuals with relatively greater value diversity exhibited greater resilience. Notably, although the resilience scale used in this study was originally developed for adolescents (Oshio et al., 2002), it was applied across a wide age range in the present research. As a supplementary analysis, we tested a model that included an interaction term between Value Diversity and age, in addition to the best-fit model. The interaction effect was not statistically significant, and the model including the interaction showed a higher BIC (15948.75) compared to the best-fit model (BIC = 15915.32). This finding suggests that the influence of Value Diversity on resilience scores, as measured by this scale, is not restricted to adolescence but may be consistently observed across different age groups.

### 3.6 Relationship between value proportion and life satisfaction

Next, we conducted a linear regression analysis to determine whether life satisfaction varied by Value Proportion, and whether the relationship differed between the US and Japan. Factors relevant to our hypotheses were the effect of Value Proportion and the interaction effect between Value Proportion and country. To control for demographic factors, we included them as covariates in the model. Because the proportion of intrapersonal, interpersonal, and extra-personal values sum to one, including all three in the model results in perfect multicollinearity. Therefore, in our analysis, in addition to the demographic covariates, we entered the proportion of interpersonal and extra-personal values, as well as their interaction terms with country (i.e., proportion of interpersonal value × country and proportion of extra-personal value × country), as fixed effects. The models for all possible combinations of fixed effects were fitted and compared in terms of the degree of fit according to the BIC.

**Figure 3:**
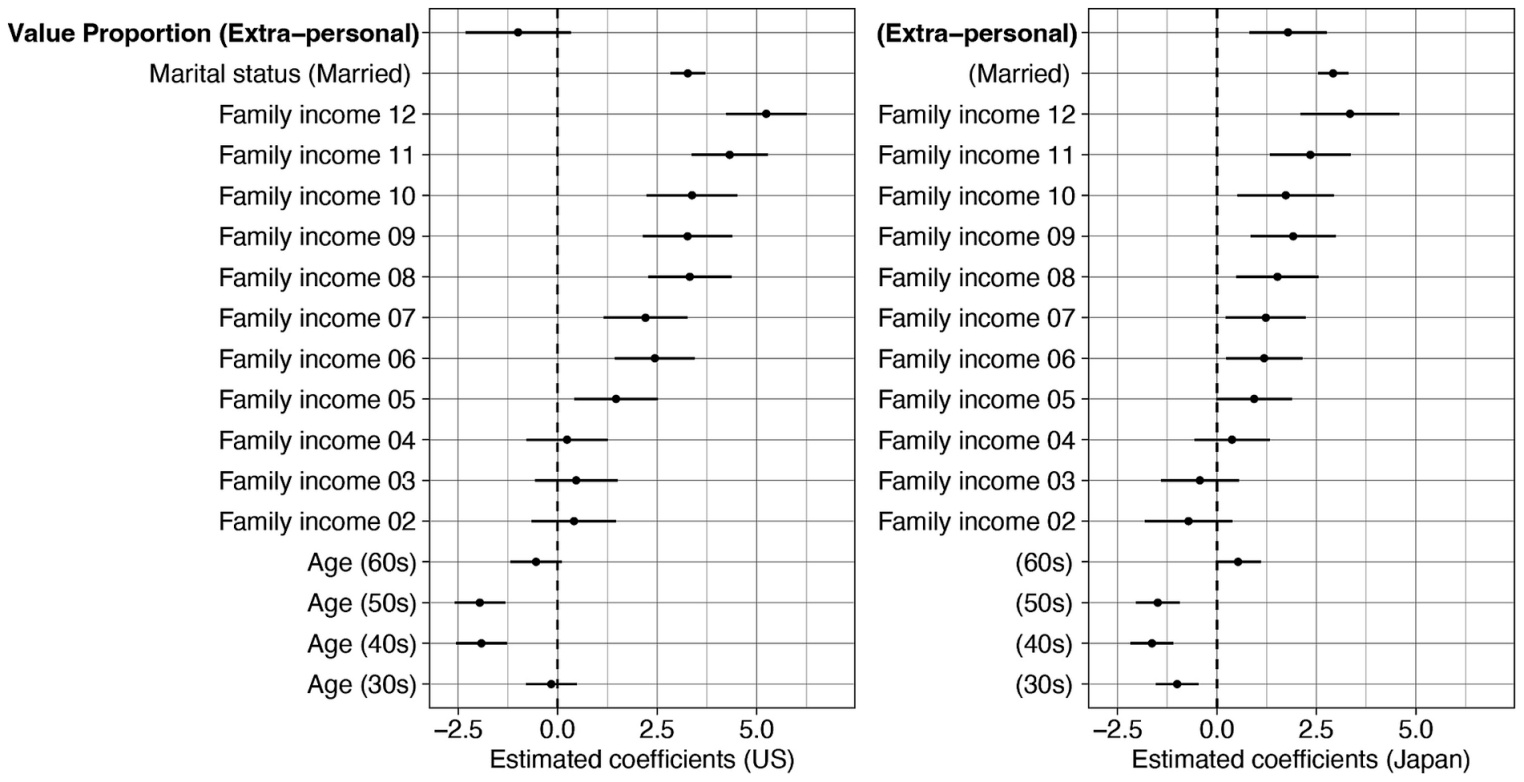
Parameter estimates of separate models for Japan and the US for life satisfaction including the proportion of extra-personal values as part of Value Proportion based on BIC and AIC. Linear regression models comparing the effects of Value Proportion, with demographic variables included as covariates. Error bars represent 95% confidence intervals of estimates.

The best-fit model contained the main effects of age, family income, marital status, country, proportion of extra-personal values, and proportion of extra-personal values × country interaction. Parameter estimates of the best-fit model are shown in Supplementary Table 4. The results suggest that the relationship between the proportion of extra-personal values and life satisfaction differs across countries. To further investigate the interaction between proportion of extra-personal values and country, we conducted post-hoc analyses by testing separate models for each country. Parameter estimates from the country-specific models are presented in Figure 3. In Japan, individuals with a higher proportion of extra-personal values tended to report greater life satisfaction (*B* = 1.788, 95%CI [0.816, 2.759], t_4800_ = 3.608, *p* < 0.001), whereas in the United States, no such relationship was observed (*B* = −0.990, 95%CI [−2.314, 0.335], t_4800_ = −1.465, *p* = 0.143). This finding suggests that the influence of Value Proportion on life satisfaction varies by country and was observed only in Japan, where individuals with a higher proportion of extra-personal values tended to show greater life satisfaction.

It should be noted that neither the model that added only the main effect of the proportion of intrapersonal values to the best-fit model (BIC = 65961.42) nor the model that included both the main effect and its interaction with country (BIC = 65970.18) showed a higher BIC than the best-fit model (BIC = 65952.21). Therefore, models including the proportion of intrapersonal values were not selected.

### 3.7 Relationship between value proportion and resilience

Finally, we conducted a linear regression analysis to determine whether resilience scores varied by Value Proportion, and whether the relationship differed between the US and Japan. The fixed effects included in the model were identical to those in the model for life satisfaction, namely, the demographic characteristics, the proportion of interpersonal and extra-personal values, and their interaction terms with country as fixed effects.

**Figure 4:**
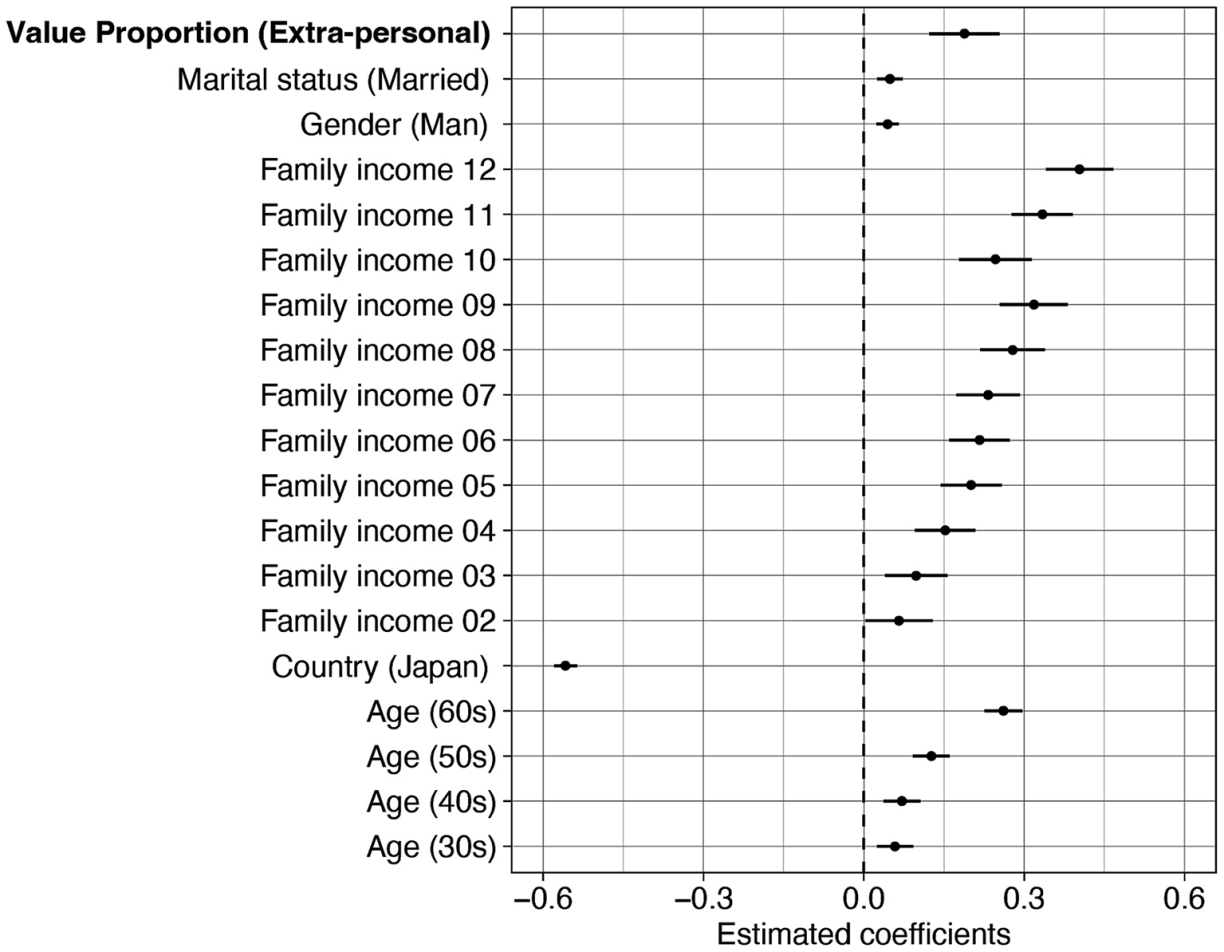
Parameter estimates of the best-fit model for resilience with the effect of Value Proportion based on BIC. Linear regression models comparing the effects of Value Proportion, with demographic variables included as covariates. Error bars represent 95% confidence intervals of estimates.

The best-fit model contained the main effects of age, gender, family income, marital status, country, and proportion of extra-personal values, but no proportion of extra-personal values × country interaction. Parameter estimates of the best-fit model are shown in Figure 4 and Supplementary Table 5. In the best-fit model, the effect for the proportion of extra-personal values (*B* = 0.188, 95%CI [0.122, 0.254], t_10017_ = 5.593, *p* < 0.001) was greater than 0. This indicates that, regardless of country, individuals with a relatively higher proportion of extra-personal values exhibited greater resilience.

It should be noted that neither the model that added only the main effect of the proportion of intrapersonal values to the best-fit model (BIC = 15928.89) nor the model that included both the main effect and its interaction with country (BIC = 15932.54) showed a higher BIC than the best-fit model (BIC = 15920.57). Therefore, models including the proportion of intrapersonal values were not selected.

### 3.8 General discussion

In this study, we examined the relationship of the balance of values with life satisfaction and resilience in a large-scale cross-cultural survey in the US and Japan, using a two-stage selection method: (1) selecting three items within the three categories (intrapersonal, interpersonal, and extra-personal values) and then (2) reselecting the most important items among the selected nine. Since there were no items with extremely high or low selection rates in either the US or Japan, the list we used in the present study seems reasonable in capturing the diverse values present among individuals in both countries. In the first selection stage, there were commonalities in values considered important across cultures, alongside some notable differences in selection rates, such as “maintaining boundaries,” which was frequently selected in Japan, and “prayer,” which was frequently selected in the US. These findings may reflect the tendency toward greater interpersonal distance in East Asian cultures (Sorokowska et al., 2017), as well as the relatively low importance of prayer in Japan, which is known for its low level of religious affiliation (WIN-Gallup International, 2012).

The research question of the second selection stage was: Is the balance of values that has been historically emphasized in East Asian culture (e.g., Swaminathan et al., 2021) particularly important in the East Asian sphere, or is this balance also relevant in the European-American cultural area, given that the WHO also points to the importance of relationships with society and the world (World Health Organization, 2022)? Our findings suggest that Value Diversity showed a consistent relationship with subjective well-being across cultures, whereas Value Proportion—particularly proportion of extra-personal values—yielded partially different results in Japan and the United States. With regard to Value Diversity, individuals with relatively greater Value Diversity exhibited greater resilience regardless of country, but this was not related to life satisfaction. Regarding Value Proportion, a positive association was found in Japan between the proportion of extra-personal values and life satisfaction, whereas no such association was observed in the US. In contrast, regardless of country, individuals with a higher proportion of extra-personal values tended to exhibit greater resilience.

Value Diversity—one aspect of the balance of values—was not associated with life satisfaction but was found to be related to resilience. This association between Value Diversity and resilience suggests that individuals who place greater importance on a diverse range of values tend to exhibit higher levels of resilience, particularly in relation to the scope of their social relationships. Furthermore, as this association did not differ between participants from Japan and the US, it appears to reflect a pattern consistent across cultures. Previous studies have demonstrated that diverse social group memberships contribute to greater resilience (Iyer et al., 2009; Jones and Jetten, 2011). In contrast to focusing on the diversity of social group memberships, the present study highlights the role of the balance and diversity of values prioritized by individuals, suggesting that placing balanced importance on values they fulfill personally, values they fulfill in their relationships with others, and values they fulfill in their relationships with society as a whole and the greater world may also be associated with greater resilience. However, the present study did not directly assess the number of social groups to which participants belonged. As such, future research is needed to clarify whether Value Diversity and the scope of social relationships independently contribute to resilience, or whether individuals with broader social ties are also more likely to place importance on a wider range of values, thereby enhancing resilience. Given that resilience reflects an individual’s capacity to recover from challenges, the observed link between Value Diversity and resilience suggests that Value Diversity, although unrelated to present life satisfaction, could play a role in enhancing life satisfaction in the future.

Value proportion, which is the other aspect of the balance of values—specifically, the proportion of extra-personal values—was associated with life satisfaction in Japan but not in the US. Considering that the proportion of extra-personal values was also higher in Japan than in the US, this finding may reflect cultural differences as noted by Uchida and Ogihara (2012). In European-American cultural contexts such as the US, individuals tend to pursue happiness as autonomous agents, placing greater importance on values they fulfill personally, such as personal goal attainment. In contrast, in East Asian cultural contexts, individuals tend to view themselves as interdependent with others and society, such that connections beyond the individual self—particularly those with society as a whole—may contribute more strongly to their well-being. However, the relationship between the proportion of extra-personal values and resilience was found to be consistent across countries, and individuals with a higher proportion of selected extra-personal values demonstrated greater resilience. Considering that the proportion of participants selecting “Prayer” was high in the US, and “Gratitude for life” was prevalent in both Japan and the US, these findings may align with previous reports suggesting a positive association between religious belief or spirituality and resilience (Fradelos et al., 2018; Javanmard, 2013; Schwalm et al., 2022).

One potential limitation of this study lies in the use of the two-stage selection method, which was intended to reduce the psychological burden on respondents. This method, in which respondents were asked to select choices within each of the three categories of intrapersonal, interpersonal, and extra-personal in the first selection stage, resulted in a larger number of respondents who selected one choice in each category (approximately 70%). In a previous free-text survey of university students in Japan (Watanabe and Chen, 2021; JST RISTEX HITE, 2019), the proportion of individuals with high intrapersonal values was greater. In future research, it might be useful to capture individual differences in the balance of values by using a method that involves less psychological burden on the respondent and does not induce a specific choice pattern. For example, all of the value items, regardless of value category, could be presented one at a time in a random order, and respondents could mark the ones they felt were somewhat important to them for later reference. Respondents would then be asked to select the final three values from the values marked at the end. In addition, it is worth noting that some values conceptually lie at the boundaries between categories. For instance, the value of “To love others” may be considered an interpersonal value when the object is family, romantic partners, or friends, but an extra-personal value when the object is humanity as a whole. In the present study, “To love others” was classified as an interpersonal value because it was assumed that respondents would most often interpret it in relation to close others. However, because such boundary-spanning values exist, future studies could improve clarity by asking respondents to specify the intended object of each value.

Another limitation of this study concerns the timing of the data collection. The survey period coincided with the COVID-19 pandemic, and in the Japanese sample, the collection of subjective well-being indicators and value-related data occurred at different time points. However, previous studies measuring life satisfaction and resilience during the COVID-19 period (Nakagawa et al., 2025; Nishimoto et al., 2023) have shown that the average levels of life satisfaction and resilience among Japanese participants remained stable between August 2020 (the first survey period in this study) and April 2021 (the second survey period). These findings suggest that these indicators are relatively stable within individuals, even during the pandemic, and thus, the impact of differences in the timing of data collection on the present results is likely to be minimal.

Nevertheless, regarding the relationship between balance of values and subjective well-being, it is important to note that while differences in the balance of values might influence well-being; the reverse causal pathway is also possible: higher levels of well-being may affect an individual’s balance of values. Therefore, causal inferences cannot be drawn from the present findings alone. Future longitudinal studies are needed to examine these causal and reciprocal relationships in more detail.

It should also be noted that while both the East Asian and European-American cultural spheres encompass a diverse range of countries, this study was conducted in only two countries—Japan and the United States. Notably, both countries have a high level of socioeconomic development. Although we found that individuals with greater Value Diversity and those who placed greater emphasis on extra-personal values tended to be more resilient across countries, it is possible that the relationship between the balance of values and resilience may be affected by socioeconomic development, given that previous studies have reported a different relationship between values and well-being in countries with high socioeconomic development compared to that in countries with low socioeconomic development (Sortheix and Lönnqvist, 2014).

While the present study examined the relationship between the balance of values and self-reported psychological resilience, it remains unclear whether the balance of values is also associated with resilience in response to actual adverse life events. Future research should address this issue by conducting longitudinal or cohort studies that continuously assess both the balance of values and experienced life events over time.

Another point that should be considered is that, in this study, we focused on long-term well-being, and utilized life satisfaction and resilience as indices of subjective well-being. However, given that a previous study (Joshanloo and Ghaedi, 2009) reported that the relationship between values and subjective well-being varies across different types of well-being (e.g., affective well-being, cognitive well-being, psychological well-being, and social well-being), it is possible that the relationship between the balance of values and well-being is different for other indices. To address these points, further research on the use of these well-being indices in different cultures may be useful.

## 4 Conclusion

In this study, rather than focusing on a specific value or the subjective “sense of balance,” we examined the “balance of values,” specifically looking at the degree to which individuals valued each of the following categories: (1) intrapersonal values (the values fulfilled within the individual), (2) interpersonal values (the values that are fulfilled in their relationships with others), and (3) extra-personal values (the values related to their connection to society and the world). We assessed two dimensions of this balance: (A) Value Diversity, defined as the extent of diversity in value selection by identifying how many of the three predefined value categories were endorsed; and (B) Value Proportion, defined as the relative emphasis placed on each value category by identifying which category was selected more frequently.

Our findings revealed that, regardless of cultural context, individuals with greater Value Diversity exhibited higher resilience, though this was not associated with life satisfaction. With respect to Value Proportion, individuals who placed greater emphasis on extra-personal values tended to demonstrate higher resilience, and in Japan, this emphasis was also associated with greater life satisfaction. Although cultural differences were observed in the relationship between Value Proportion and life satisfaction, the overall pattern suggests that individuals who consider that individual values and values related to connections with others, society, and the world are similarly important—and particularly those who emphasize connections with society and the world—are more likely to “bounce back from difficulties.” These findings indicate that, in addition to considering specific values or the subjective “sense of balance,” it is also important to take into account the balance of multiple values within an individual to enhance future well-being.

## Data Availability

The datasets presented in this study can be found in online repositories. The names of the repository/repositories and accession number(s) can be found below: Open Science Framework (OSF) [https://osf.io/6g85d/?view_only=70332f0c01d148369d7a7fe35cdd8f10].
