# Supplementary material for: The balance of intrapersonal, interpersonal, and extra-personal values and its relationship to life satisfaction and resilience in Japan and the United States
Source: Front Psychol. 2025 Jul 30;16:1606618. doi: 10.3389/fpsyg.2025.1606618 (PMC12344499; doi:10.3389/fpsyg.2025.1606618)
Supplement: Supplementary file 1 [file Supplementary_file_1.pdf]

Supplementary Material

1 Supplementary Methods Table

Supplementary Table 1. Intrapersonal, interpersonal, and extra-personal values used in the survey.

	Values	Description of each value in English	Description of each value in Japanese
	**Category A: Intrapersonal values**		
1	**Immersion in interests**	To immerse myself in the things I like and enjoy.	好きなことや楽しいことに没頭すること
2	**Goal-oriented**	To work proactively toward my goals.	自らの目標をもって前向きに行動すること
3	**Hope**	To find hope for the future.	未来に希望を見出すこと
4	**Tenacity**	To be able to bounce back even if I encounter a difficult or uncomfortable situation.	嫌なことや辛いことがあっても気持ちを切り替えられること
5	**Keeping your own pace**	To live at my own pace without worrying about what is happening around me or the opinions of others.	周りで起きていることや他人の目を気にせずマイペースでいること
6	**Mindfulness**	To be at peace and not to be swayed by my emotions.	自分の感情に振り回されず、心が穏やかな状態であること
7	**Self-awareness**	To have the time to consider and face what I want to do and what I should do.	自分が何をしたいのか、何をすべきなのかを考え、向き合う時間をもつこと
8	**Self-acceptance**	To accept my own failings and weaknesses.	自分の悪いところや嫌なところも含めて、受け入れること
9	**Self-determination**	To decide for myself how I will act.	自分で決めて行動すること
10	**Sense of competence**	To feel that I am capable.	自分に能力があると感じられること
11	**Sense of accomplishment**	To feel a sense of achievement.	達成感を感じられること
12	**Personal growth**	To feel that I have grown.	自分が成長したと感じられること
13	**Challenging oneself**	To challenge myself.	挑戦すること
14	**Fulfilling one's potential**	To demonstrate the capabilities and skills that I have attained.	自分に備わっている能力や技術を発揮すること
15	**Vitality**	To be full of motivation and energy.	やる気や活力に満ちた状態であること
16	**Uncompromisingness**	To take things on without compromise.	妥協せずに物事に取り組むこと
17	**Curiosity**	To take things on with curiosity and interest.	好奇心や興味をもって物事に取り組むこと
18	**Error avoidance**	As far as possible, not to make any conspicuous mistakes.	人目に付くような失敗をできるだけしないこと
19	**To be the best**	To aim for the top.	一番を目指すこと
20	**Uniqueness**	To have an individual identity that is different from others.	他者と異なる独自性があること
	**Category B: Interpersonal values**		
1	**Close relationships**	To have good relationships with those close to me (e.g., family, friends, significant others, etc.)	自分にとって身近な人（家族・友人・恋人など）との関係が良好なこと
2	**Community relations**	To have positive interpersonal relationships in the communities to which I belong.	自分が属しているコミュニティの中で人間関係が良好なこと
3	**Shared experiences with family/friends**	To talk, share experiences, eat together, or otherwise spend time together with those close to me (family, friends, significant others, etc.).	自分にとって身近な人（家族・友人・恋人など）と話をしたり、体験を共有したり、食事をするなど、一緒に時間を過ごすこと

4	**Shared experiences with the community**	To talk, share experiences, eat together, or otherwise spend time together with people in the communities to which I belong.	自分が属しているコミュニティの人たちと話をしたり、体験を共有したり、食事をするなど、一緒に時間を過ごすこと
5	**Building new relationships**	To form new connections with people.	人との新しいつながりを作ること
6	**Cooperation**	To cooperate with others and achieve things through mutual encouragement.	他者と協力し、励まし合い何かを成し遂げること
7	**Achieving victory**	To win against the competition.	競争に勝つこと
8	**Harmony**	To consider the positions of other people and not to disrupt the sense of harmony.	それぞれの人の立場を考慮して調和を乱さないこと
9	**Acceptance**	To be accepted by others and to feel like I have found the right place for me.	人から受け入れられ、自分の居場所があると感じられること
10	**Self-esteem**	To feel like I have value as a person in the eyes of others.	自分が他者にとって価値がある人間であると感じられること
11	**Altruism**	To help people who are in trouble.	困っている人のために何かをすること
12	**Trust**	To have relationships of mutual trust.	信頼し合える関係性があること
13	**To love others**	To love.	愛すること
14	**To be loved**	To be loved by family, friends, significant others, etc.	友人や家族、恋人などから愛されること
15	**Mutual gratitude**	To have relationships of mutual gratitude.	感謝し合える関係性があること
16	**Having good role models**	To know people who are good examples and role models.	尊敬する人やロールモデルがいること
17	**To be respected**	To be treated with respect by those around me.	周りの人から敬意をもって接してもらえること
18	**Mutual empathy**	To have relationships with mutual empathy.	共感し合える関係性があること
19	**To have fun with everyone**	To have a good time with everyone.	みんなで一緒に盛り上がること
20	**Maintaining boundaries**	To have a good sense of distance from others.	他人とよい距離感をとれていること
21	**Shared Values**	To have relationships with shared values.	価値観を共有できる関係性があること
22	**Leadership**	To demonstrate leadership and to lead the way for others.	リーダーシップを発揮して周りの人を導くこと
	**Category C: Extra-personal values**		
1	**Trustworthy society**	To belong to a trustworthy society.	信頼できる社会に属していること
2	**Peace**	For there to be world peace.	世界が平和であること
3	**Diversity**	To understand and accept that people are different from each other.	人はそれぞれ違うということを理解し、受け入れること
4	**Contributing to society**	To contribute to the society.	社会に貢献できること
5	**Role in Society**	To believe that there is a role for me in the society.	社会の中で自分の役割があると思えること
6	**Following social norms**	To follow the standards and rules of the society.	社会の規範やルールを守ること
7	**Acknowledging the unknown**	To be aware that an unknown world exists.	自分の知らない世界があることを認識すること
8	**Being a part of nature**	To feel connected to the nature.	自然とのつながりを感じること
9	**Gratitude for life**	To have a feeling of gratitude for being alive.	生きていることへの感謝の気持ちをもつこと
10	**Awareness of mortality**	To feel that life has an end.	命が有限であることを感じること
11	**Awareness of human kindness**	To be aware that kindness is a quality of all people.	だれもが優しさをもっていることに気がつくこと

12	**Balancing conflicting views**	To find the best path for different parties even if there are differences in standpoint and opinion.	立場や思想が対立しても、両方にとってよい道を探すこと
13	**Prayer**	To pray.	祈りを捧げること
14	**Observe religious precepts**	To behave according to religious precepts and standards.	宗教的な戒律や規範を守って行動すること
15	**Equitable society**	To belong to an equitable society.	公平な社会に属していること
16	**Ancestral bonds**	To care for long-lasting relationships, such as with forebears and descendants.	祖先や子孫など、時間を超えたつながりを大事にすること

Note. The English version of the text was created by requesting a double back-translation from Crimson Interactive Japan Co., Ltd.

Supplementary Table 2. Demographic characteristics of participants from the US and Japan.

Demographic variable		US (n = 4818)	Japan (n = 5219)
Gender	Woman	2355 (51.1%)	2638 (49.5%)
	Man	2463 (48.9%)	2581 (50.5%)
Age	20s	894 (18.6%)	991 (19.0%)
	30s	955 (19.8%)	1033 (19.8%)
	40s	963 (20.0%)	1060 (20.3%)
	50s	1015 (21.1%)	1058 (20.3%)
	Over 60s (The oldest is 79)	991 (20.6%)	1077 (20.6%)
Family income	Less than \$10K (¥1M)	297 (6.2%)	202 (3.9%)
	\$10K to \$20K (¥1M to ¥2M)	341 (7.1%)	270 (5.2%)
	\$20K to \$30K (¥2M to ¥3M)	381 (7.9%)	527 (10.1%)
	\$30K to \$40K (¥3M to ¥4M)	414 (8.6%)	740 (14.2%)
	\$40K to \$50K (¥4M to ¥5M)	368 (7.6%)	708 (13.6%)
	\$50K to \$60K (¥5M to ¥6M)	454 (9.4%)	698 (13.4%)
	\$60K to \$70K (¥6M to ¥7M)	370 (7.7%)	501 (9.6%)
	\$70K to \$80K (¥7M to ¥8M)	387 (8.0%)	405 (7.8%)
	\$80K to \$90K (¥8M to ¥9M)	292 (6.1%)	335 (6.4%)
	\$90K to \$100K (¥9M to ¥10M)	273 (5.7%)	193 (3.7%)
	\$100K to \$150K (¥10M to ¥15M)	718 (14.9%)	466 (8.9%)
	More than \$150K (¥15M)	523 (10.9%)	174 (3.3%)
Marital status	Unmarried (Including widowed, separated, or divorced)	2322 (51.8%)	2451 (53.0%)
	Married	2496 (48.2%)	2768 (47.0%)

Note. Factors that are reference classes in the linear mixed model are shaded in gray. Number of individuals for each attribute in each country is shown in digits, with the percentage in parentheses.

2 Supplementary Results

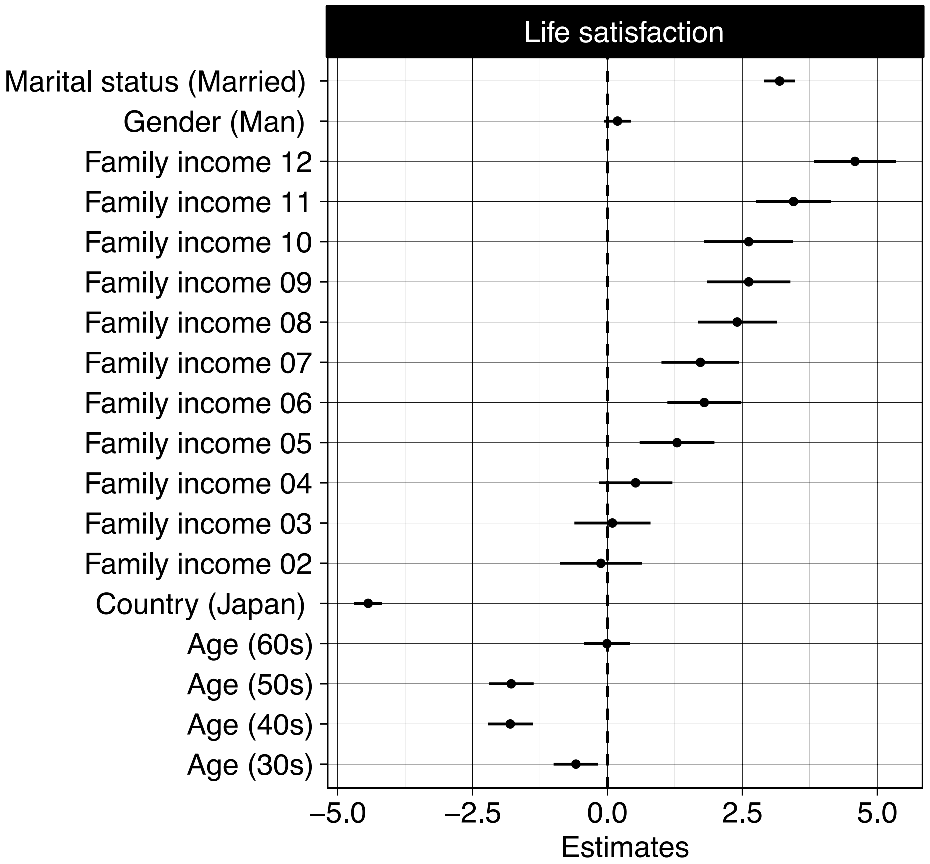

Supplementary Figure 1. Parameter estimates of the linear regression model for life satisfaction.

The reference class is defined as people living in the United States, in their 20s, unmarried, women, and with a family income of less than $10K (¥1M). Error bars represent 95% confidence intervals of estimates.

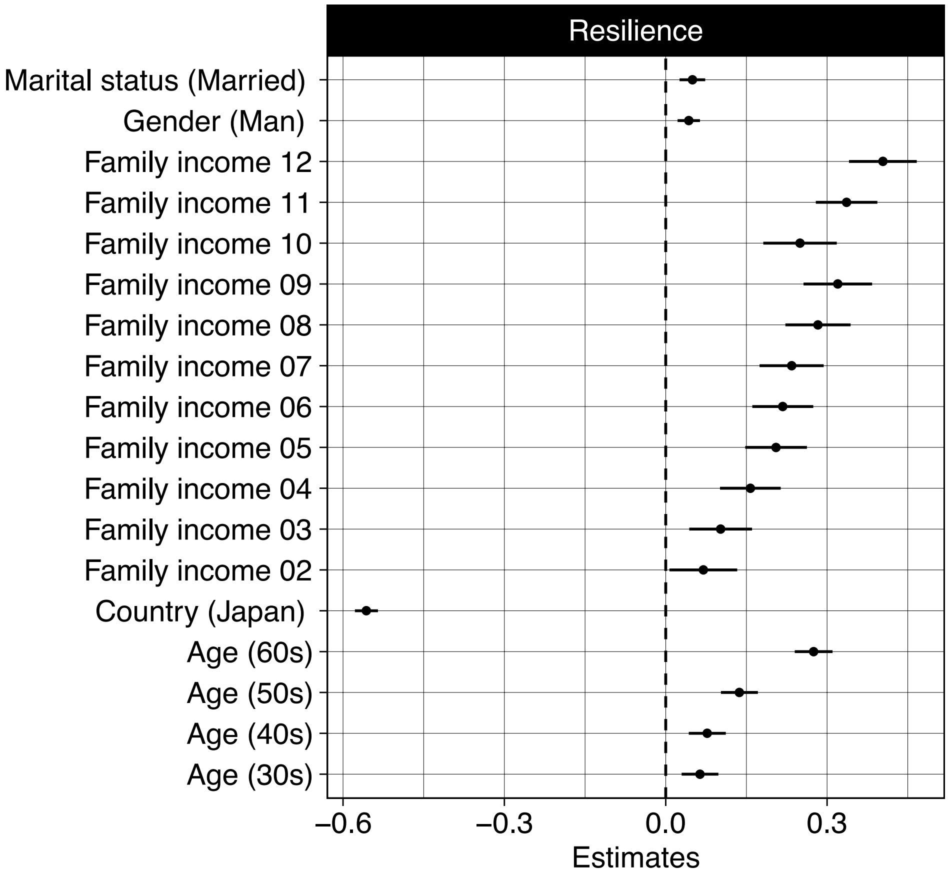

Supplementary Figure 2. Parameter estimates of the linear regression model for the Adolescent Resilience Scale.

The reference class is defined as people living in the United States, in their 20s, unmarried, women, and with a family income of less than $10K (¥1M). Error bars represent 95% confidence intervals of estimates.

Supplementary Table 3. Parameter estimates of the best-fit model for resilience with the effect of Value Diversity based on BIC and AIC.

		Coefficient (B)	*Lower CI*	*Upper CI*	*SE*	*Standardized Coefficient (β)*	*t value*	*p value*
(Intercept)		3.071	3.002	3.141	0.035	-0.130	86.878	<0.001
Country	Japan	-0.551	-0.573	-0.530	0.011	-0.890	-50.403	<0.001
Gender	Man	0.042	0.021	0.062	0.011	0.070	3.922	<0.001
Age	30s	0.059	0.024	0.093	0.017	0.090	3.359	<0.001
	40s	0.071	0.036	0.105	0.018	0.110	4.023	<0.001
	50s	0.127	0.093	0.162	0.018	0.200	7.256	<0.001
	60s	0.264	0.228	0.299	0.018	0.420	14.650	<0.001
Family income	$10K to $20K (¥1M to ¥2M)	0.067	0.004	0.130	0.032	0.110	2.082	0.037
	$20K to $30K (¥2M to ¥3M)	0.099	0.040	0.157	0.030	0.160	3.319	<0.001
	$30K to $40K (¥3M to ¥4M)	0.152	0.095	0.208	0.029	0.240	5.276	<0.001
	$40K to $50K (¥4M to ¥5M)	0.203	0.145	0.260	0.029	0.330	6.939	<0.001
	$50K to $60K (¥5M to ¥6M)	0.215	0.159	0.272	0.029	0.350	7.454	<0.001
	$60K to $70K (¥6M to ¥7M)	0.229	0.170	0.289	0.030	0.370	7.543	<0.001
	$70K to $80K (¥7M to ¥8M)	0.278	0.218	0.339	0.031	0.450	9.011	<0.001
	$80K to $90K (¥8M to ¥9M)	0.319	0.255	0.383	0.032	0.510	9.824	<0.001
	$90K to $100K (¥9M to ¥10M)	0.245	0.177	0.314	0.035	0.390	7.055	<0.001
	$100K to $150K (¥10M to ¥15M)	0.333	0.275	0.390	0.029	0.530	11.407	<0.001
	More than $150K (¥15M)	0.403	0.340	0.466	0.032	0.650	12.554	<0.001
Marital Status	Married	0.049	0.026	0.073	0.012	0.080	4.067	<0.001
Value Diversity		0.057	0.038	0.075	0.009	0.050	6.045	<0.001

Adjusted R^2: 0.28, *BIC*: 15915.32, *AIC*: 15763.82

BIC for the full model: 15924.53, *AIC* for the full model: 15765.82

Note. The reference class is defined as people living in the United States, in their 20s, unmarried, women, and with a family income of less than $10K (¥1M). CI indicates 95% confidence intervals of the estimates. SE indicates standard errors.

Supplementary Table 4. Parameter estimates of the best-fit model for life satisfaction with the effect of Value Proportion based on BIC and AIC.

		Coefficient (B)	*Lower CI*	*Upper CI*	*SE*	*Standardized Coefficient (β)*	*t value*	*p value*
(Intercept)		20.212	19.523	20.902	0.352	-0.040	57.471	<0.001
Country	Japan	-5.402	-5.950	-4.855	0.279	-0.610	-19.342	<0.001
Age	30s	-0.583	-0.996	-0.169	0.211	-0.080	-2.764	0.006
	40s	-1.792	-2.209	-1.376	0.213	-0.250	-8.431	<0.001
	50s	-1.796	-2.213	-1.379	0.213	-0.250	-8.438	<0.001
	60s	-0.033	-0.461	0.395	0.219	-0.005	-0.152	0.879
Family income	$10K to $20K (¥1M to ¥2M)	-0.088	-0.850	0.674	0.389	-0.010	-0.227	0.821
	$20K to $30K (¥2M to ¥3M)	0.103	-0.602	0.808	0.360	0.010	0.287	0.774
	$30K to $40K (¥3M to ¥4M)	0.525	-0.157	1.207	0.348	0.070	1.508	0.132
	$40K to $50K (¥4M to ¥5M)	1.320	0.627	2.012	0.353	0.180	3.736	<0.001
	$50K to $60K (¥5M to ¥6M)	1.824	1.140	2.508	0.349	0.250	5.225	<0.001
	$60K to $70K (¥6M to ¥7M)	1.757	1.037	2.478	0.367	0.240	4.784	<0.001
	$70K to $80K (¥7M to ¥8M)	2.450	1.717	3.182	0.374	0.340	6.556	<0.001
	$80K to $90K (¥8M to ¥9M)	2.653	1.883	3.422	0.393	0.370	6.756	<0.001
	$90K to $100K (¥9M to ¥10M)	2.652	1.827	3.477	0.421	0.370	6.303	<0.001
	$100K to $150K (¥10M to ¥15M)	3.500	2.809	4.191	0.353	0.480	9.928	<0.001
	More than $150K (¥15M)	4.617	3.857	5.377	0.388	0.640	11.910	<0.001
Marital Status	Married	3.175	2.887	3.463	0.147	0.440	21.631	<0.001
Value Proportion	Extra-personal value	-1.140	-2.379	0.100	0.632	-0.030	-1.803	0.071
Value Proportion × Country	Japan × Extra-personal value	3.182	1.584	4.781	0.815	0.070	3.903	<0.001

Adjusted R^2: 0.22, *BIC*: 65952.21, *AIC:* 65800.72

BIC for the full model: 65993.72, *AIC for the full model*: 65806.15

Note. The reference class is defined as people living in the United States, in their 20s, unmarried, women, and with a family income of less than $10K (¥1M). CI indicates 95% confidence intervals of the estimates. SE indicates standard errors.

Supplementary Table 5. Parameter estimates of the best-fit model for resilience with the effect of Value Proportion based on BIC.

		Coefficient (B)	*Lower CI*	*Upper CI*	*SE*	*Standardized Coefficient (β)*	*t value*	*p value*
(Intercept)		3.168	3.114	3.222	0.028	-0.130	115.208	<0.001
Country	Japan	-0.558	-0.579	-0.537	0.011	-0.900	-51.121	<0.001
Gender	Man	0.044	0.024	0.065	0.011	0.070	4.179	<0.001
Age	30s	0.058	0.024	0.093	0.017	0.090	3.353	<0.001
	40s	0.071	0.037	0.106	0.018	0.110	4.054	<0.001
	50s	0.126	0.091	0.160	0.018	0.200	7.156	<0.001
	60s	0.261	0.226	0.297	0.018	0.420	14.463	<0.001
Family income	\$10K to \$20K (¥1M to ¥2M)	0.066	0.003	0.129	0.032	0.110	2.050	0.040
	\$20K to \$30K (¥2M to ¥3M)	0.098	0.040	0.157	0.030	0.160	3.305	<0.001
	\$30K to \$40K (¥3M to ¥4M)	0.152	0.096	0.208	0.029	0.240	5.281	<0.001
	\$40K to \$50K (¥4M to ¥5M)	0.201	0.144	0.258	0.029	0.320	6.872	<0.001
	\$50K to \$60K (¥5M to ¥6M)	0.217	0.160	0.273	0.029	0.350	7.500	<0.001
	\$60K to \$70K (¥6M to ¥7M)	0.233	0.173	0.292	0.030	0.370	7.655	<0.001
	\$70K to \$80K (¥7M to ¥8M)	0.278	0.218	0.339	0.031	0.450	9.005	<0.001
	\$80K to \$90K (¥8M to ¥9M)	0.318	0.254	0.381	0.032	0.510	9.785	<0.001
	\$90K to \$100K (¥9M to ¥10M)	0.246	0.178	0.314	0.035	0.400	7.074	<0.001
	\$100K to \$150K (¥10M to ¥15M)	0.334	0.277	0.391	0.029	0.540	11.446	<0.001
	More than \$150K (¥15M)	0.404	0.341	0.467	0.032	0.650	12.587	<0.001
Marital Status	Married	0.049	0.025	0.073	0.012	0.080	4.023	<0.001
Value Proportion	Extra-personal values	0.188	0.122	0.254	0.034	0.050	5.593	<0.001

Adjusted R^2: 0.28, *BIC*: 15920.57

BIC for the full model: 15940.9

Note. The reference class is defined as people living in the United States, in their 20s, unmarried, women, and with a family income of less than \$10K (¥1M). CI indicates 95% confidence intervals of the estimates. SE indicates standard errors

2.1 Effect of value types on resilience controlling for Schwartz's basic values

To examine whether the effects of the balance of values—Value Diversity and Value Proportion—identified in the present study remain significant even after controlling for established value constructs, we included Schwartz's ten basic values as covariates in supplementary analyses. These values—Power, Achievement, Hedonism, Self-direction, Universalism, Stimulation, Benevolence, Tradition, Conformity, and Security—were measured using questionnaire items from the sixth round of the World Values Survey (Inglehart et al., 2014). Participants responded on a six-point scale ranging from 1 (not at all like me) to 6 (very much like me). For Benevolence, which was assessed with two items, the mean score (Cronbach's alpha = 0.83) was used, while a single-item score was used for each of the other values.

To examine whether the relationship between Value Diversity and Resilience observed in the present study remains significant after controlling for Schwartz's ten basic values (Schwartz, 1992), we conducted a linear regression analysis in which the scores for Power, Achievement, Hedonism, Self-Direction, Universalism, Stimulation, Benevolence, Tradition, Conformity, and Security were added as covariates to the best-fit model (Figure 2, Supplementary Table 3). In the model, the effect of Value Diversity (β= 0.019, 95%CI [0.002, 0.035], t_{10007} = 2.235, p = 0.025) were greater than 0. This suggests that, even controlling for Schwartz's ten basic values, individuals with greater Value Diversity exhibited higher resilience.

In the same way, to examine the relationship between Value Proportion and Resilience, we conducted a linear regression analysis in which the scores for Schwartz's values were added as covariates to the best-fit model (Figure 4, Supplementary Table 5). The results showed that the effect of the proportion of extra-personal values were greater than 0 (β= 0.063, 95%CI [0.004, 0.122], t10007 = 2.107, p = 0.035). Thus, even after controlling for Schwartz's ten basic values, the effect of placing greater emphasis on extra-personal values on resilience was observed.

Since the relationship between Value Proportion and life satisfaction was observed only in Japan, we conducted a linear regression analysis in which the scores for Schwartz's values were added as covariates to the model that examined the effect of the proportion of extra-personal values in Japan (Figure 3). The results showed that the effect of the proportion of extra-personal values remained positive (β= 0.063, 95%CI [0.004, 0.122], t10007 = 2.107, p = 0.035), indicating that even after controlling for Schwartz's ten basic values, the effect of placing greater emphasis on extra-personal values on life satisfaction was observed in Japan.

2.2 Results of model selection based on AIC

As a supplementary analysis, we conducted model-selection based on the Akaike Information Criterion (AIC; Akaike, 1974), which is often used even with relatively small sample sizes. For the model for resilience with the effect of Value Diversity and the model for life satisfaction with the effect of Value Proportion, the same models were identified as the best-fit models under both the AIC and BIC criteria (see Supplementary Table 3, Supplementary Table 4).

In the model for resilience with the effect of Value Proportion, the best-fit model based on the AIC criterion (Supplementary Table 6) included an interaction between country and the proportion of extra-personal values, in addition to the best-fit model identified using the BIC criterion (see Supplementary Table 5). To further examine this interaction, we conducted post-hoc analyses by

testing separate models for each country. In the US, individuals with a higher proportion of extra-personal values tended to report greater resilience (β= 0.259, 95%CI [0.163, 0.354], $t_{4799} = 5.293$, $p < 0.001$). In Japan, a similar relationship was observed, although the effect was slightly smaller than in the US (β= 0.134, 95%CI [0.043, 0.224], $t_{5200} = 2.892$, $p = 0.004$). The confidence intervals for the effect of the proportion of extra-personal values in the Japanese model overlapped with those in the US model, suggesting no substantial difference between the two countries.

Supplementary Table 6. Parameter estimates of the best-fit model for resilience with the effect of Value Proportion based on AIC.

		Coefficient(B)	*Lower CI*	*Upper CI*	*SE*	*Standardized Coefficient (β)*	*t value*	*p value*
(Intercept)		3.146	3.088	3.204	0.030	-0.130	106.195	<0.001
Country	Japan	-0.517	-0.562	-0.472	0.023	-0.900	-22.376	<0.001
Gender	Man	0.045	0.024	0.066	0.011	0.070	4.245	<0.001
Age	30s	0.058	0.023	0.092	0.017	0.090	3.299	<0.001
	40s	0.070	0.036	0.105	0.018	0.110	3.986	<0.001
	50s	0.125	0.090	0.159	0.018	0.200	7.089	<0.001
	60s	0.260	0.225	0.296	0.018	0.420	14.395	<0.001
Family income	$10K to $20K (¥1M to ¥2M)	0.064	0.001	0.127	0.032	0.100	1.986	0.047
	$20K to $30K (¥2M to ¥3M)	0.097	0.039	0.156	0.030	0.160	3.272	0.001
	$30K to $40K (¥3M to ¥4M)	0.151	0.095	0.207	0.029	0.240	5.251	<0.001
	$40K to $50K (¥4M to ¥5M)	0.199	0.142	0.257	0.029	0.320	6.818	<0.001
	$50K to $60K (¥5M to ¥6M)	0.216	0.159	0.272	0.029	0.350	7.463	<0.001
	$60,K to $70K (¥6M to ¥7M)	0.231	0.171	0.290	0.030	0.370	7.596	<0.001
	$70K to $80K (¥7M to ¥8M)	0.276	0.216	0.337	0.031	0.440	8.934	<0.001
	$80K to $90K (¥8M to ¥9M)	0.316	0.253	0.380	0.032	0.510	9.741	<0.001
	$90K to $100K (¥9M to ¥10M)	0.244	0.176	0.312	0.035	0.390	7.019	<0.001
	$100K to $150K (¥10M to ¥15M)	0.332	0.274	0.389	0.029	0.530	11.369	<0.001
	More than $150K (¥15M)	0.403	0.340	0.466	0.032	0.650	12.570	<0.001
Marital Status	Married	0.049	0.025	0.073	0.012	0.080	4.032	<0.001
Value Proportion	Extra-personal values	0.268	0.166	0.371	0.052	0.070	5.128	<0.001
Value Proportion × Country	Japan × Extra-personal value	-0.135	-0.267	-0.003	0.067	-0.030	-2.004	0.045

Adjusted R^2: 0.28, *AIC*: 15767.05

AIC for the full model: 15767.76

Note. The reference class is defined as people living in the United States, in their 20s, unmarried, women, and with a family income of less than $10K (¥1M). CI indicates 95% confidence intervals of the estimates. SE indicates standard errors.

3 References

Akaike, H. (1974). A new look at the statistical model identification. IEEE Transactions on Automatic Control, 19(6), 716–723. https://doi.org/10.1109/TAC.1974.1100705

Inglehart, R., Haerpfer, C., Moreno, A., Welzel, C., Kizilova, K., Diez-Medrano, J., Lagos, M., Norris, P., Ponarin, E., and Puranen, B. et al. (eds.). (2014). World Values Survey: Round Six - Country-Pooled Datafile Version. Madrid: JD Systems Institute.

Schwartz, S.H. (1992). Universals in the content and structure of values: Theoretical advances and empirical tests in 20 countries. Advances in Experimental Social Psychology, 25, 1–65. https://doi.org/10.1016/S0065-2601(08)60281-6
